# Compatibility of *Serratia ureylitica* Su_YN1, Malaria Transmission-Blocking Bacterium, with the *Anopheles aquasalis* Vector

**DOI:** 10.3390/tropicalmed10090249

**Published:** 2025-08-31

**Authors:** Marília Andreza da Silva Ferreira, Elen Sabrina dos Reis Martins, Ricardo de Melo Katak, Keillen Monick Martins Campos, Elerson Matos Rocha, Rosemary Aparecida Roque, Pritesh Jaychand Lalwani, Luciete Almeida Silva, Edson Júnior do Carmo, Paulo Paes de Andrade, Sibao Wang, Luciano Andrade Moreira, Marcelo Jacobs-Lorena, Claudia María Ríos-Velásquez

**Affiliations:** 1 Programa de Pós-Graduação em Biologia da Interação Patógeno—Hospedeiro, Instituto Leônidas e Maria Deane—ILMD/Fiocruz Amazônia, Manaus 69057-070, Brazil; marilia.andreza.masf@gmail.com (M.A.d.S.F.); emartins@aluno.fiocruz.br (E.S.d.R.M.); 2 Unidade Acadêmica de Ciências Biológicas, Centro de Saúde e Tecnologia Rural—Universidade Federal de Campina Grande, Patos 58708-110, Brazil; 3 Laboratório em Ecologia de Doenças Transmissíveis na Amazônia, Instituto Leônidas e Maria Deane—ILMD/Fiocruz Amazônia, Manaus 69057-070, Brazil; ricardokatak@hotmail.com (R.d.M.K.); keillen.monick@gmail.com (K.M.M.C.); 4 Laboratório de Malária e Dengue, Instituto Nacional de Pesquisas da Amazônia, Manaus 69057-070, Brazil; elerson.matos13@gmail.com (E.M.R.); rosebio1996@yahoo.com.br (R.A.R.); 5 Laboratório de Diagnóstico e Controle de Doenças Infecciosas na Amazônia, Instituto Leônidas e Maria Deane—ILMD/Fiocruz Amazônia, Manaus 69057-070, Brazil; pritesh.lalwani@fiocruz.br; 6 Laboratório Diversidade Microbiana da Amazônia com Importância para a Saúde, Instituto Leônidas e Maria Deane—ILMD/Fiocruz Amazônia, Manaus 69057-070, Brazil; luciete.silva@fiocruz.br; 7 Centro de Apoio Multidisciplinar, Universidade Federal do Amazonas, Manaus 69057-070, Brazil; ejcarmo.biotec@gmail.com; 8 Target DNA Soluções em Biotecnologia Ltda, Curitiba 83810-000, Brazil; andrade@ufpe.br; 9 Key Laboratory of Insect Developmental and Evolutionary Biology, CAS Center for Excellence in Molecular Plant Sciences, Institute of Plant Physiology and Ecology, Chinese Academy of Sciences, Shanghai 200032, China; sbwang@cemps.ac.cn; 10 Laboratório Mosquitos Vetores: Endossimbiontes e Interação Patógeno-Vetor, Instituto René Rachou—IRR/Fiocruz Minas Gerais, Belo Horizonte 30510-010, Brazil; luciano.andrade@fiocruz.br; 11 Johns Hopkins Malaria Research Institute, Bloomberg School of Public Health, Baltimore, MD 21229, USA

**Keywords:** *Anopheles aquasalis*, mosquito microbiota, mosquito fitness, malaria

## Abstract

Malaria continues to affect millions of people around the world. Current control strategies have not been sufficient, and additional tools are required for malaria elimination. A promising approach is the use of bacteria from the mosquito microbiota, such as the Su_YN1 *Serratia ureilytica* bacterium, which is shown to strongly impair the development of *P. falciparum* and *P. berghei* in *Anopheles* mosquitoes. To evaluate the potential of using *S. ureilytica* Su_YN1 to block the *Plasmodium* parasite in South American vectors, we investigated its effects on mosquito fitness and survival. We found that this bacterium does not affect the longevity, blood feeding, fecundity and fertility of *Anopheles aquasalis*, an important South American vector. Overall, our results provide baseline support for the potential implementation of Su_YN1 for the control of malaria transmission in South America.

## 1. Introduction

Malaria affects more than 263 million people and kills more than half a million people every year, mostly African children under 5 years old [1]. In 2023, over half a million cases were reported in the Americas, most of them in the Amazon region. It is concerning that since 2015, hardly any progress has been made to reduce malaria deaths [1]. This means that the available tools to fight malaria are insufficient, and new strategies must urgently be implemented.

When a susceptible mosquito bites a person infected with *Plasmodium*, it ingests gametocytes and becomes infected. In the insect’s midgut, the gametocytes are rapidly converted to male and female gametes. After fertilization, zygotes differentiate into mobile ookinetes that cross the intestinal epithelium to give rise to oocysts. Each oocyst gives rise to thousands of sporozoites that are released into the hemocoel and, from there, migrate to and invade the salivary glands, where they remain for the rest of the mosquito’s life [2,3,4]. The low oocyst number (about five in field mosquitoes) represents a bottleneck to parasite development in the mosquito and affects their formation, which is a crucial step for blocking *Plasmodium* transmission [5].

Some gut bacteria, such as *Comamonas* sp., *Acinetobacter* sp., *Pantoea* sp., *Serratia marcescens* and *Elizabethkingia anophelis*, among others, have shown a reduction in the *Plasmodium falciparum* oocyst load in *Anopheles gambiae* [6,7,8].

The mosquito’s intestinal microbiota affects the transmission and development of *Plasmodium* and can modulate the immunity of the mosquito vector [9]. The microbiota interfere with the development of the parasite by stimulating the insect’s immune response and producing metabolites that affect the survival of the pathogen [10]. In a study comparing vector species (*An. coluzzii* and *An. arabiensis)* with non-vector species (*An. quadriannulatus*), it was found that there are differences in relation to susceptibility to the parasite. Non-vector species are more tolerant of the microbiota and refractory to the parasite, while vector species display the opposite behavior [11].

In particular, *Delftia tsuruhatensis* [12] and *Serratia ureilytica* Su_YN1 (referred to as YN1) [13] are two mosquito microbiome bacteria that naturally (not genetically modified) strongly block the development of *Plasmodium* in mosquitoes. As such, they provide much promise as a new tool for malaria control.

YN1 was isolated from wild *Anopheles sinensis* from the Yunnan Peninsula in China, where the local malaria cases are lower than in other geographical regions. YN1 strongly inhibits the development of the human parasite *P. falciparum* and the rodent parasite *Plasmodium berghei* in *Anopheles stephensi* and *Anopheles gambiae* by the secretion of anti-*Plasmodium* lipase [13]. Importantly, YN1 was shown to be able to spread through mosquito populations [13]. Evaluating the potential fitness cost of a bacterium is a crucial property that needs to be considered for its potential use in the control of vector-transmitted diseases.

*Anopheles aquasalis* is an important malaria vector in coastal regions of Brazil [14] and South America [15]. It can be easily maintained as laboratory colonies and has been shown to be a good model for *Plasmodium*–*Anopheles* interaction studies [16,17]. Given the YN1 potential for use in malaria transmission, we evaluated the compatibility of this bacterium with the *A. aquasalis* vector, as it is not its native bacterium. We found that this bacterium is completely compatible with *A. aquasalis*, as mosquito longevity, blood-feeding rate, fecundity and fertility were not affected by the presence of YN1. These findings support the potential use of YN1 to fight malaria in South America.

## 2. Materials and Methods

### 2.1. Anopheles aquasalis

*A. aquasalis* mosquitoes were provided by the insectary of the Ecologia de Doenças Transmissiveis na Amazônia Laboratory, Instituto Leônidas & Maria Deane, Manaus, Amazonas, Brazil. This mosquito colony has been maintained in the laboratory since 2009.

The larvae were reared in plastic containers measuring 40 cm × 12 cm × 4 cm at a density of 100 larvae per tray. The larvae were fed with crushed and sieved TetraMin Flakes. After emerging, the adults were kept in insect cages. Adult mosquitoes were maintained with 10% sucrose ad libitum in a 12 h/12 h day/night light cycle at 27–28 °C and 70–80% relative humidity.

### 2.2. Bacteria

Two bacteria species were used: *Pantoea agglomerans* [18], used as a mosquito symbiotic bacterium control, and *Serratia ureylitica* Su_YN1 (YN1) [13]. The bacteria YN1 were cultured overnight and electroporated in the Electroporator 2510 equipment (Eppendorf^®^, Hamburg, Germany), exposed to a voltage of 1900 volts. Electrocompetent YN1 bacteria [16] were transformed with the plasmid pUC57-18k_GFP-HasA (3501 pb). The GFP gene sequence used in this study was prospectively identified in silico in the GenBank (NCBI) database, originating from the cloning vector pBAD-GFPuv (Accession No. U62637.1). A gene expression cassette containing the constitutive PnptII promoter, E-Tag, HasA and a terminator was then designed in silico, along with the GFP sequence. These elements were cloned into the pUC57 vector synthesized by GenOne (Brazil). The resulting plasmid was named pUC57-18k_GFP-HasA [19,20,21]. Colonies expressing green fluorescent protein were identified by exposure to UV light (Supplementary Figure S1).

### 2.3. Bacterial Culture and Introduction into Mosquitoes

YN1 and *P. agglomerans* were cultured overnight in an LB broth containing 50 μg/mL kanamycin and 100 μg/mL carbenicillin, centrifuged, washed three times in sterile 1x PBS and resuspended in sterile 5% (wt/vol) sucrose solution to a final 1 × 10^7^ cell/mL^−1.^

Cotton pads were soaked with a bacterial suspension of YN1 and *P. agglomerans* (1 × 10^7^ cell/mL) in a sterile 5% (wt/vol) sucrose solution and given to the adult mosquitoes. After 36 h, the bacterial cotton pads were replaced with new sterile cotton pads soaked in the 5% (wt/vol) sterile sucrose solution and given to the mosquitoes for two additional days. Control mosquitoes were fed exclusively with the 5% (wt/vol) sterile sucrose solution. Following feeding with YN1, 100 µL of the bacteria suspension obtained from the cotton pads was plated into LB plates and checked for fluorescence 24 h later. Positive fluorescence indicates the presence of bacteria YN1 in the cotton.

### 2.4. Effects of Different Concentrations of YN1 on the Survival of A. aquasalis

Females of *A. aquasalis* were fed with YN1 concentrations of 1 × 10^5^ cell/mL^−1^, 1 × 10^6^ cell/mL^−1^ and 1 × 10^7^ cell/ml^−1^ and a sterile sucrose solution as a control. Groups of 50 females from each treatment were separated and checked daily for mortality for 15 d. The experiments were repeated three times independently.

### 2.5. Effects of YN1 on the Survival of Female and Male A. aquasalis

After bacteria feeding, groups of 50 females and 50 males from each treatment (YN1, *P. agglomerans* and the control) were separated and checked daily for mortality, totaling 150 females and 150 males for each biological replicate. This experiment was performed three independent times for a total of 900 mosquitoes.

### 2.6. Effects of YN1 on Mosquito Blood Feeding, Fecundity and Fertility

Mosquitoes were fed with bacteria YN1 and *Pantoea*, and two days later, they were provided with a blood meal. The number of engorged and non-engorged mosquitoes was counted. Four days after a blood meal, 50 females from each treatment were placed in 15 mL Falcon tubes (one female/tube). The Falcon tubes had their walls covered with filter paper, and the bottom of each tube was filled with 7.5 mL of water to stimulate female oviposition and capped with cotton pads. The number of eggs laid by each female was counted after two days. The eggs were placed in a basin containing pure water, and the hatched larvae were counted at the L2 larval stage. The eggs were placed in a basin measuring 40 cm × 12 cm × 4 cm containing 600 mL of tap water.

All experiments were performed three independent times.

### 2.7. Statistical Analysis

To analyze the effect of the bacteria on mosquito longevity, Kaplan–Meier survival analysis was performed, and *p*-values were defined by the log-rank test (Mantel–Cox). To evaluate the effect on the mosquito blood feeding, fecundity and fertility, we used the Kruskal–Wallis test with Dunn’s multiple comparisons, with a significance threshold of 0.05. All data analyses were performed using the GraphPad Prism v.6 software (San Diego, CA, USA, https://www.graphpad.com/).

### 2.8. Ethical Statements

The experiments with bacteria were carried out at the Laboratory of Infectious Diseases and Immunology of the Postgraduate Program in Basic and Applied Immunology—PPGIBA from the Federal University of Amazonas, approved with Biosafety Level II by the National Technical Commission for Biosafety—CTNBio (CQB 095/98, technical decision No. 8714/2023). The project was registered in the National System for the Management of Genetic Heritage and Associated Traditional Knowledge (SisGen, access code A5B26D1). Blood used for mosquito feeding was collected from volunteers, and the procedure was approved by the Research Ethical Committee (CEP UEA 6.718.957).

## 3. Results

### 3.1. Effect of Different YN1 Concentrations on the Survival of A. aquasalis

**Figure 1:**
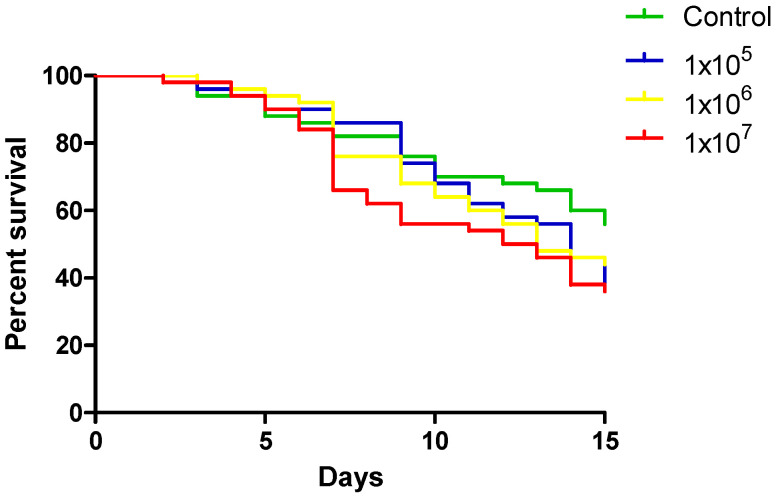
The effect of different concentrations of YN1 on the survival of *A. aquasalis*. Newly emerged adult female mosquitoes were fed for 36 h on a cotton pad moistened with a 5% sugar solution containing 10^5^ cells/mL^−1^, 10^6^ cells/mL^−1^ or 10^7^ cells/ml^−1^-YN1 bacterium and a 5% sterile sucrose solution as control. Mosquito survival was monitored daily for 15 days. Survival rates were not significantly different among different bacteria concentrations (log-rank, df = 3, *p* = 0.2293). The values of three independent experiments were combined.

The survival of *A. aquasalis* females fed with different YN1 concentrations was evaluated. Three independent replicates were performed, each one with 50 individuals, totaling 150 females. There was no statistical difference among the analyzed concentrations (log-rank, df = 3, *p* = 0.2293) (Figure 1).

We conclude that YN1 does not affect mosquito survival. The dose used for the remaining experiments was standardized to 1 × 10^7^ cell/mL^−1^.

### 3.2. Effect of YN1 on Adult Male and Female A. aquasalis Survival

Adult female and male *A. aquasalis* mosquitoes were fed with bacteria for 36 h and then monitored daily for mortality until the last individual in each group died. Three independent biological replicates were performed, with 50 males and 50 females for each experiment, for a total of 900 mosquitoes.

**Figure 2:**
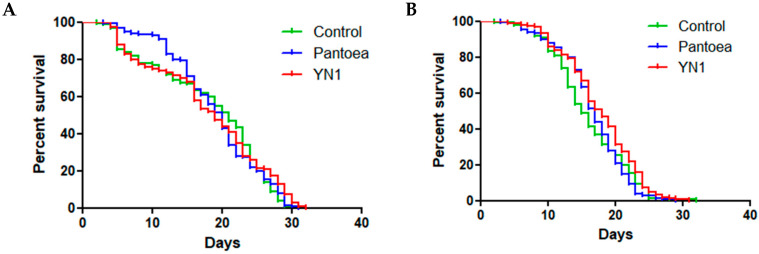
Survival of female (**A**) and male (**B**) *A. aquasalis* fed with YN1 bacteria (red line), with control *P. agglomerans* bacteria (blue line) or with a control 5% sterile sucrose solution (green line). Survival rates were not significantly different among different treatments of females (log-rank, df = 2, *p* = 0.6088), but they were significantly different among the treatments of males (log-rank, df = 2, *p* = 0.0442) using the Mantel–Cox test. The values of three independent experiments were combined.

In the female group, approximately 50% of the mosquitoes died by the 20th day after being exposed to the treatments. The female mosquitoes survived for 30 days in all. Importantly, there was no significant difference among treatments of the female mosquitoes (log-rank, df = 2, *p* = 0.6088) (Figure 2A).

In the male group, however, there was a significant difference (log-rank, df = 2, *p* = 0.0442) (Figure 2B).

We conclude that YN1 does not affect female mosquito survival.

### 3.3. Effects of YN1 on Mosquito Blood-Feeding Behavior

**Figure 3:**
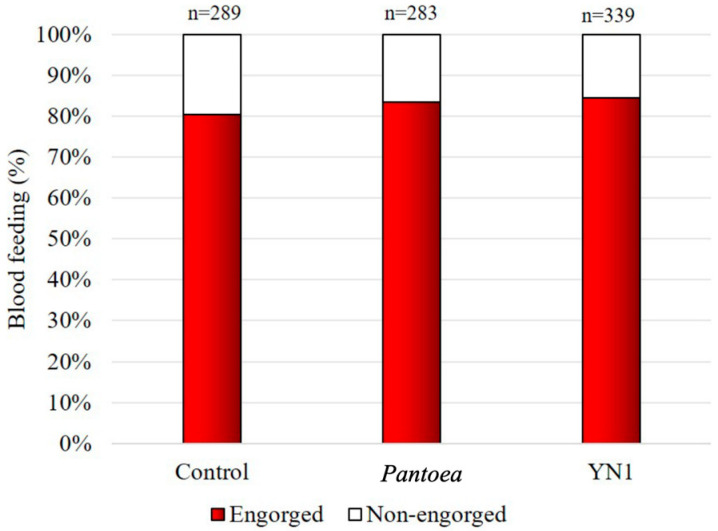
Effect of *Serratia* YN1 on *A. aquasalis* blood-feeding behavior. Mosquitoes were provided with sterile sugar (control), *Pantoea* bacteria (a control symbiotic bacterium) or YN1 bacteria for 36 h and were then allowed to feed on blood for 2 h. The proportion of mosquitoes that fed on blood is indicated by the red bars. The number of females assayed is displayed on the top of each bar. There was no statistical difference among the three experimental setups (ANOVA *p* = 0.9536). The values of three independent experiments were combined.

To investigate whether YN1 affects blood-feeding behavior, female mosquitoes were provided with cotton balls soaked in sugar alone, with a *Pantoea* bacteria suspension in sugar (*Pantoea* was used as a mosquito symbiotic bacterium control), or with a YN1 bacteria suspension in sugar. This was followed by providing a blood meal using glass feeders. As shown in Figure 3, there was no impact of YN1 on *A*. *aquasalis* blood-feeding behavior (ANOVA *p* = 0.9536).

### 3.4. Effects of YN1 on Mosquito Fecundity

**Figure 4:**
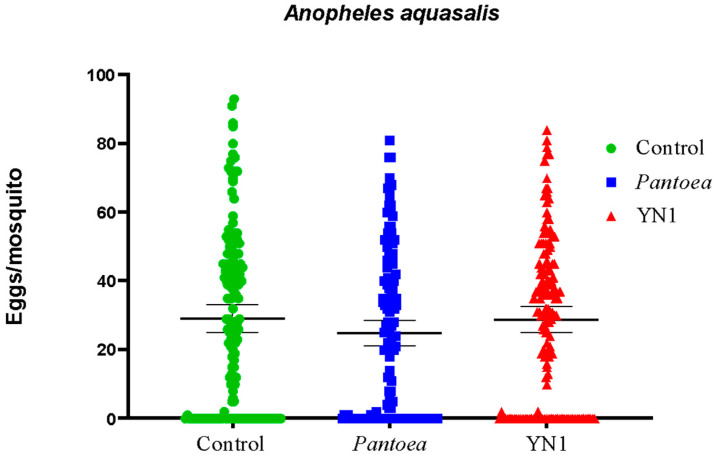
The effect of *Serratia* YN1 on *A. aquasalis* fecundity. Mosquitoes were provided with sterile sugar (control), *Pantoea* bacteria or YN1 bacteria for 36 h, followed by a blood meal. Eggs were collected 4 d later and counted. Each dot represents the number of eggs laid by a single female mosquito. Horizontal lines represent medians. There was no significant difference among treatments (*p* = 0.3593) using the Kruskal–Wallis test. The values of three independent experiments were combined, using the eggs from a total of 450 mosquitoes.

*A. aquasalis* mosquitoes were provided with cotton balls soaked with sugar alone, a *Pantoae* bacteria suspension in sugar or a YN1 bacteria suspension in sugar. After providing a blood meal, the pregnant females were separated for oviposition. The eggs were collected on filter papers from individual females and counted. The median number of eggs per female laid was 29 for the control group, 25 for the *Pantoea*-fed females, and 30 for the YN1-fed females (Figure 4), and there were no significant differences among the groups (Kruskal–Wallis test, *p* = 0.3593). We conclude that YN1 bacteria do not impact *A*. *aquasalis* female fecundity.

### 3.5. Effects of YN1 on Mosquito Fertility

**Figure 5:**
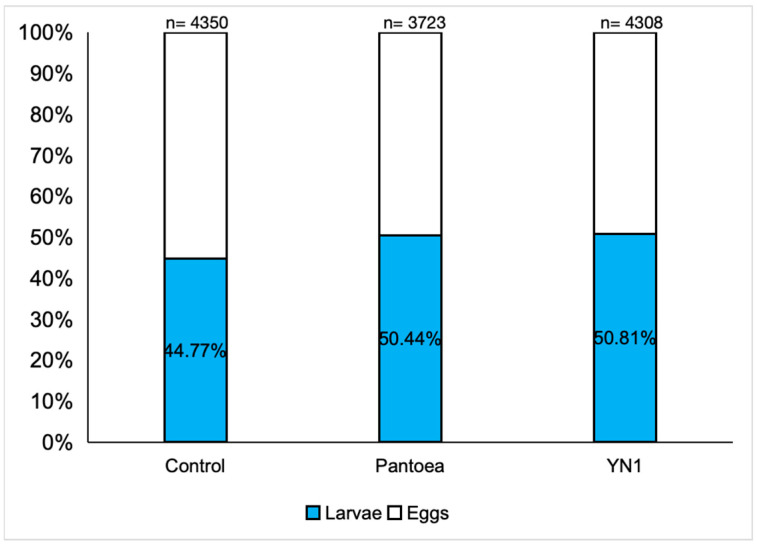
The effect of *Serratia* YN1 on *A. aquasalis* fertility. Mosquitoes were provided with sterile sugar (control), *Pantoea* bacteria or YN1 bacteria for 36 h, followed by a blood meal. Eggs were collected 4 d later, and the proportion of these eggs that hatched into larvae was counted. The number of eggs assayed is indicated on the top of each bar. There was no significant difference among treatments using ANOVA (*p* = 0.9623). The values of three independent experiments were combined.

This set of experiments was conducted to investigate the impact of the YN1 bacteria on the viability of eggs laid by female mosquitoes. *A. aquasalis* were provided with cotton balls soaked with sugar alone, a *Pantoea* bacteria suspension in sugar or a YN1 bacteria suspension in sugar. After providing a blood meal, the eggs were collected and reared in water, and the proportion of these eggs that developed into L2 larvae was determined. For the control group, 44.8% out of 4350 eggs hatched into larvae; for the *Pantoea* group, 50.4% out of 3723 eggs hatched into larvae; and for the YN1 group, 50.8% out of 4308 eggs hatched into larvae. There were no significant differences among the groups (ANOVA *p* = 0.9623) (Figure 5). We conclude that YN1 bacteria do not impact *A*. *aquasalis* female fertility.

## 4. Discussion

To date, only insecticides have been used in the form of insecticide-impregnated bed nets and indoor residual spraying to suppress transmission of the malaria parasite. These approaches have been effective and were, in great part, responsible for the pronounced decrease in malaria deaths between 2000 and 2015 [1]. However, effectiveness has decreased significantly, mainly due to the development of mosquito insecticide resistance and behavioral changes. As a result, little gain in mortality has occurred between 2015 and 2023 [1]. The development of non-insecticide-based approaches to combat malaria transmission is highly desirable.

A promising new approach involves the use of symbiotic bacteria that kill early stages of *Plasmodium* development in the mosquito midgut, thus blocking its transmission to humans. YN1, originally an *A. sinensis* gut symbiont, has been shown to effectively suppress *Plasmodium* development in various anopheline species [13]. As such, it has great potential as a tool for blocking malaria transmission. Considering the potential to translate these laboratory findings into field-based malaria control, two YN1 key properties are that the bacterium is not genetically modified and it can spread through mosquito populations via horizontal and vertical transmission from one generation to the next. As an initial step to evaluate the potential of YN1 for combating malaria transmission in the Americas, we evaluated the impact of YN1 on *A. aquasalis* survival, blood-feeding behavior, fecundity and fertility.

We found that the bacteria YN1 does not affect any of the *A. aquasalis* parameters evaluated for females, even though this bacterium originated from a different continent. *A. aquasalis* males showed a slightly higher survival when fed with YN1, which may favor dispersal and competitiveness in nature. The egg-hatching rate was between 44 and 50% in the groups evaluated. Larvae were counted at the L2 stage, avoiding manipulation of L1, and there is natural mortality within the biological cycle. In these experiments, water with iodized salt was not used, which may interfere with the larval development of the species.

Other bacteria of the genus *Serratia*, such as *Serratia marcescens* [22] and *Serratia* AS1, were also shown to have no negative effects on the fitness of *A. gambiae, A. stephensi* and *Culex pipiens* [23,24,25].

## 5. Conclusions

Our findings suggest that the YN1 bacterium does not affect mosquito fitness. Additional studies are needed to determine its ability to spread through Amazonian mosquitoes in the field and evaluate YN1’s capability of thwarting *Plasmodium* in its vector.

## Data Availability

All the data are presented in the manuscript. There is no additional data to share.

## Supplementary Materials

The following supporting information can be downloaded at: https://www.mdpi.com/article/10.3390/tropicalmed10090249/s1, Figure S1: *Serratia ureilytica* Su_YN1 transformed with GFP protein.
