# Supplementary material for: Compatibility of Serratia ureylitica Su_YN1, Malaria Transmission-Blocking Bacterium, with the Anopheles aquasalis Vector
Source: Trop Med Infect Dis. 2025 Aug 31;10(9):249. doi: 10.3390/tropicalmed10090249 (PMC12474516; doi:10.3390/tropicalmed10090249)

Supplementary data for: Compatibility of *Serratia ureylitica* Su_YN1, Malaria Transmis-sion-Blocking Bacterium, with the *Anopheles aquasalis* Vector

Supplementary Figure S1: *Serratia ureilytica* Su_YN1 transformed with GFP protein.

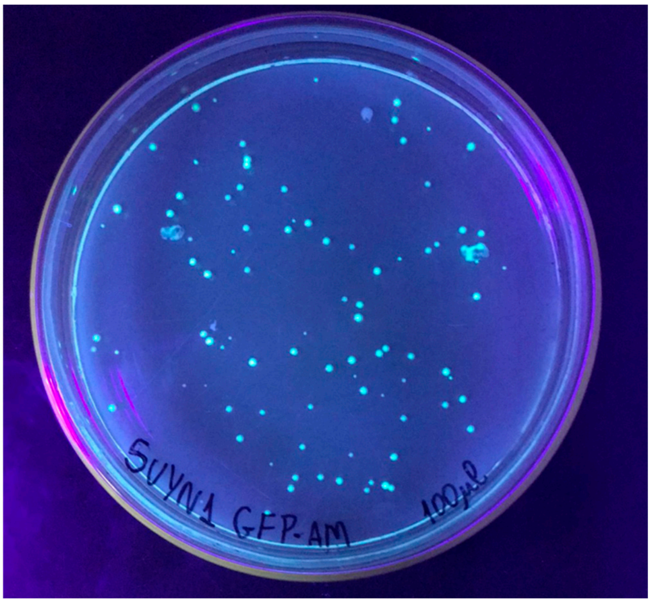

Supplement: Supplementary file 1 [file tropicalmed-10-00249-s001.zip › tropicalmed-3657183-supplementary.pdf]
